# A retrospective observational insight into COVID-19 exposures resulting from personal protective equipment (PPE) breaches

**DOI:** 10.1371/journal.pone.0268582

**Published:** 2022-05-17

**Authors:** Ujjwala Nitin Gaikwad, Oshrika Bose, Abhishek Padhi, Atul Jindal, Keshao Nagpure, Anudita Bhargava, Padma Das

**Affiliations:** 1 Department of Microbiology, All India Institute of Medical Sciences, Raipur, Chhatisgarh, India; 2 Department of Pediatrics, All India Institute of Medical Sciences, Raipur, Chhatisgarh, India; 3 Department of General Medicine, All India Institute of Medical Sciences, Raipur, Chhatisgarh, India; University of Calgary, CANADA

## Abstract

**Background:**

Healthcare workers (HCWs) stand the risk of acquiring infection directly, while attending to patients or indirectly while handling and testing patient specimens. Considering this, the present study was planned to assess Personal Protective Equipment (PPE) breaches and exposures among HCWs working in COVID-19 wards/ screening areas and to evaluate their COVID-19 positivity rates post-exposure concerning the level of exposure, type of PPE breach, and the cadre of HCWs exposed in COVID-19 wards.

**Methods:**

This retrospective cross-sectional study involved the analysis of all instances of PPE breaches which occurred during a period of nine months from June 2020 to February 2021 at a tertiary care level hospital in Central India. The analysis included all exposures involving any cadre of HCWs that occurred while handling the patients or while doffing the contaminated PPE in COVID -19 wards.

**Results:**

A total of 347 PPE breaches were analyzed from the available records of the Hospital Infection Control team repository. Amongst the 347 breaches, 268 (77.2%) were classified as low-risk exposures and 79 (22.8%) as high-risk exposures. Cadre wise distribution of high and low-risk exposures revealed that, PPE breaches occurred most commonly in the category of nursing officers (n = 174, 50.1%). Among all of the breaches, 15.2% of high-risk exposures and 2.6% of low-risk exposures resulted in COVID-19 positivity with a cumulative positivity of 5.4%. Collectively, non-mask related breaches accounted for the majority (63.2%) of the positive COVID-19 cases.

**Conclusion:**

Appropriate use of PPE by HCWs is vital for their protection. However, breaches in the use of PPE may occur while managing COVID-19 patients due to physical and mental exhaustion among HCWs resulting from work overload. Early identification and appropriate management of HCWs with high-risk exposures can help prevent transmission to other hospital staff and patients, thus preserving resources and workforce.

## Introduction

The cluster of pneumonia cases that had its origin in Wuhan, China has culminated into a full-blown pandemic causing severe morbidity and mortality [1]. Coronavirus disease 2019 now known by the eponym COVID-19 is caused by severe acute respiratory syndrome virus-2 (SARS-CoV-2) [2]. Until 26^th^ June 2021, COVID-19 had already caused 1,81,186,023 Cases and 3925227 deaths [3].

Healthcare workers (HCWs) consist of all paid and unpaid persons serving in healthcare settings who have the potential for direct or indirect exposure to patients or their infectious materials, including body substances (e.g., blood, tissue, and specific body fluids); contaminated medical supplies, devices, and equipment; contaminated environmental surfaces; or contaminated air. HCWs consist of but are not limited to doctors, nurses, medical laboratory scientists, maintenance staff, clinical trainees, volunteers, emergency medical service personnel, nursing assistants, home healthcare personnel, technicians, therapists, phlebotomists, pharmacists, dental healthcare personnel, students and trainees and contractual staff [4]. They are at risk of acquiring infection directly, while attending to patients, or indirectly while handling and testing patient specimens [5]. Specimens having potential risk to cause infection are respiratory secretions or saliva of the infected persons in the form of droplets or aerosols that are likely to be produced during aerosol-generating procedures or when a patient coughs/sneezes [6]. Hence, there is a need for proper infection control practices for the prevention of infection to these individuals for the proper functioning of the healthcare system as to avoid occupational exposure and dissemination of infection. Since, HCWs deal directly with COVID-19 patients, their safety should be an urgent focus in the global response to the pandemic [7].

Personal protective equipment (PPE) offers considerable protection against occupational exposure to SARS-CoV-2 in addition to the basic infection prevention measures including hand hygiene and distancing. Recommendations regarding appropriate usage of PPE have been issued by national and international organizations like the World Health Organization (WHO), Centre for Disease Control (CDC) and the India Ministry of Health and Family Welfare (MOHFW) which have also been updated from time to time. Based upon the latter recommendations, every health care facility has also formulated guidelines for proper use of PPE with standardized donning and doffing protocols in COVID-19 areas to minimize the chances of acquiring infection. HCWs have also been provided regular training on the correct techniques of donning and doffing of PPE.

However, even after abiding with the recommended PPE protocol, while working with COVID-19 patients, an accidental breach in the protection may occur. This may happen during emergencies or during routine care due to various factors including improper methods of donning and doffing of PPE, poor visibility due to fogging of eye goggles and suboptimal quality of PPE. Such breaches need to be assessed thoroughly to decide upon the management of exposed HCWs. As per WHO as well as MOHFW recommendations, HCWs with high-risk exposures require monitoring for the development of infection and quarantine followed by testing for SARS- CoV -2 to prevent further transmission. This approach is directed towards preserving the workforce required to preserve essential services. On the other hand, many of the exposures may not always lead to infection. Considering this, it is important to review the nature and extent of PPE breaches that have occurred during COVID-19 patient care, to identify the most common types/ patterns of breaches that occurred and their significance in causing SARS-CoV-2 positivity among HCWs.

This information will help to facilitate PPE use protocols and will generate evidence that can be shared during training and sensitization programs in-house as well as with peer institutions to devise/modify the preventive strategies. Accordingly, the present study was planned to assess the PPE breaches/ exposures among HCWs working in COVID-19 wards/ screening areas and to evaluate their COVID positivity rates post-exposure concerning the level of exposure, type of PPE breach, and the cadre of HCWs exposed in COVID-19 wards.

## Materials and methods

This retrospective cross-sectional study involved the analysis of all instances of PPE breaches which occurred during a period of nine months from June 2020 to February 2021 at a tertiary care level hospital in Central India. The analysis included all exposures involving any cadre of HCWs that occurred while handling the patients or while doffing contaminated PPE in COVID-19 wards. Only those exposures which were noticed by or reported to the infection control team were included in the study. Exposures due to interaction with a positive case outside the COVID wards or any kind of community exposures were excluded from the study.

The COVID-19 area of our hospital is basically a three tier system. There is a screening zone where suspected patients are screened for COVID-19. The positive patients are admitted into the COVID-19 ward and any seriously ill patients are shifted to the COVID-19 ICU of our hospital.

In our hospital, all HCWs working in COVID wards/ICUs are required to wear the following PPE: N95 mask, goggles/face shield, cap, coverall with hood, two pairs of gloves, and shoe covers. Detailed recommendations can be found in S1 File.

All HCWs prior to their posting at COVID wards were provided with obligatory hands-on training on PPE donning and doffing (including N95 fit checking) by the hospital’s infection control staff. On-site hand-holding sessions were also held for HCWs who were having trouble donning and doffing their PPE. Furthermore, instructive videos on donning and doffing of full and individual PPE were developed and distributed to all healthcare staff.

The hospital has a well-defined mechanism to observe and report PPE breaches/ incidents of potential exposures occurring on COVID wards and the rational use of PPE as advised by the MOHFW is thoroughly followed [8]. Monitoring occurs on a round the clock basis i.e., 24 hours per day and 7 days per week, by the infection control nursing officers (ICNOs) posted in the COVID-19 area under the Hospital Infection Control Program of the hospital. To describe briefly, breaches/exposures occurring inside the wards or at the time of doffing of PPE are immediately reported to the ICNOs posted on each floor of the COVID wards. The ICNOs advise immediately on spot corrective action if applicable to the case and note down the details of an incident of breach/exposure in a predesigned proforma. The incident is then reported to the faculty in charge of the ward and the Infection Control Officer assesses the risk level involved in the exposure (Table 1). The exposures are then categorized as ’Low risk’ or ’High risk’ as per the guidelines by CDC and MOHFW [4, 9].

- **High-risk exposure**:
  - HCW or other person providing care to a COVID-19 case or lab worker handling respiratory specimens from COVID-19 cases without recommended PPE or with a possible breach of PPE
  - HCWs performing aerosol-generating procedures without appropriate PPE.
  - HCWs without mask/face-shield/goggles:
  ○ having face to face contact with a COVID-19 case within 1 meter for more than 15 minutes
  ○ having accidental exposure to body fluids or having unprotected direct contact with the infectious secretions from a patient with COVID-19 or a contaminated patient care environment.
- **Low-risk exposure:**
  Contact with a person with COVID-19 having not met criteria for high-risk exposure (e.g., brief interactions with COVID-19 patients in the hospital).

**Table 1:** Risk categorization of PPE breaches according to PPE component.

PPE Component involved	Nature of anticipated risk	Low risk	High risk
Gloves (two pairs)	Accidental exposure to body fluids or having unprotected direct contact with the infectious secretions from a patient with COVID-19 or contaminated patient care environment.	• Removal of OR tears in outer gloves only with or without hand hygiene following. Removal/ tear in both pairs of gloves which was followed by hand hygiene	• Accidental removal of OR tear in both pairs of gloves which was not followed by hand hygiene Patient care without gloves and hand hygiene. • eedlestick injuries^#^
N 95 mask	Having face to face contact with a COVID-19 case within 1 meter for more than 15 minutes	• Any exposure where expected fit of N95 mask was compromised due to accidental loosening of headbands or sudden displacement of the mask within 1-meter distance of a COVID-19 positive patient for less than 15 minutes in routine patient care settings	• Exposure where expected fit of a N95 mask was compromised because of loosening of headbands or sudden displacement of the mask within a one-meter distance for > 15 minutes during routine patient care. OR for any duration during *aerosol-generating procedures (AGPs) done on a patient.
Goggles	Having face to face contact with COVID-19 case within 1 meter for more than 15 minutes	Any exposure with loosening/removal of the goggles/face shield within 1-meter distance of a COVID-19 positive patient for less than 15 minutes in routine patient care settings	• Any exposure with loosening/ removal of the goggles/ face shield within one-meter distance for > 15 minutes during routine patient care OR for any duration during AGPs which are being carried out on a patient
Coverall	Accidental exposure to body fluids or having unprotected direct contact with the infectious secretions from a patient with COVID-19 or contaminated patient care environment.	Any cut/tear in the coverall/ slipping off of hood during routine patient care/ AGP followed by shower and change of clothes	Any cut/tear in the coverall or slipping off of the hood during routine patient care/ AGP not followed by shower and change of clothes

#Needle stick injuries were labelled as high-risk exposures based on risk assessment template provided by CDC [10].

* **List of AGPs performed in COVID ward—**Suctioning of the nasopharyngeal secretions (because closed suction is not available all the time), laryngoscopy, endotracheal intubation, any kind of airway handling like putting on Non-invasive ventilator, putting on non-rebreather mask (NRBM), putting on high-flow nasal cannula (HFNC), cardiopulmonary resuscitation.

All the HCWs posted in COVID-19 wards were provided with residence within dedicated rooms within the hospital’s campus, as some of them were hesitant to go back to their homes in off-duty hours. If they had high-risk exposures during duty, they were immediately quarantined and tested for SARS-CoV-2 on or after 7 days of exposure or if they became symptomatic as per the hospital’s policy. Until tested negative, HCWs observed quarantine and continued to do so till 14 days post exposure. Those HCWs with low-risk exposures on the other hand were not strictly quarantined and were allowed to continue working while self-monitoring their symptoms and residing in the institutional residential facility even after the exposure. The Infection Control team followed up on the HCWs who had high-risk exposures for their COVID-19 testing status after 7 days. Those who tested positive were advised to get clinical care after consultation with doctors posted on the COVID-19 wards. HCWs with low-risk exposure were tested for COVID-19 if they experienced symptoms after 7 days, or voluntarily even if they were asymptomatic after 7 days for fear of testing positive. In such cases, laboratory records were used to determine the status of COVID-19 positivity.

The study involved evaluation of all such exposures that had occurred during the study period retrospectively through review of records on breach incidents. Reports on COVID-19 positivity among the HCWs were retrieved from the records possessed by the Infection Control team and Microbiology laboratory.

### Statistical analysis

The data were collected and tabulated in an Excel (Microsoft Inc.) sheet. The data were analyzed for proportions of high and low-risk exposures concerning types of PPE breach and type of HCW cadre affected. Appropriate statistical analysis was done applying appropriate statistical tests using SPSS version 22.0 software.

### Ethics statement

The study was approved by the Institute Ethics Committee review board of the All India Institute of Medical Sciences Raipur, India. The study was conducted according to the principles expressed in the Declaration of Helsinki. (Ethics Approval number:1704/IEC–AIIMS RPR/2021).

## Results

**Fig 1:**
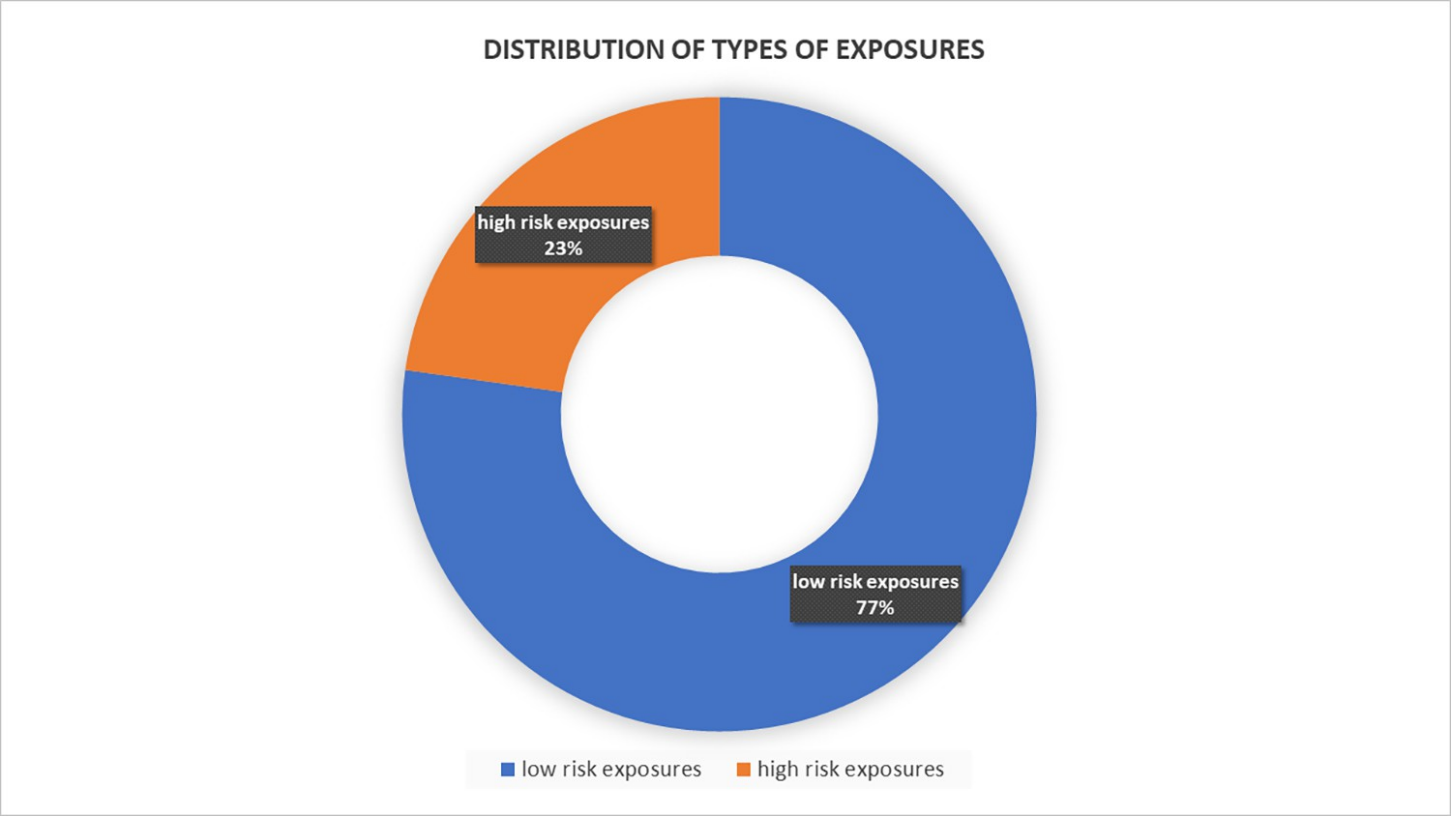
Distribution of types of exposures.

This retrospective study was carried out over nine months spanning from June 2020 to February 2021. A total of 347 PPE breaches were analysed from the available records of the Hospital Infection Control team repository with 268 (77.2%) breaches classified as low-risk exposures and 79 (22.8%) breaches classified as high-risk exposures (Fig 1).

**Fig 2:**
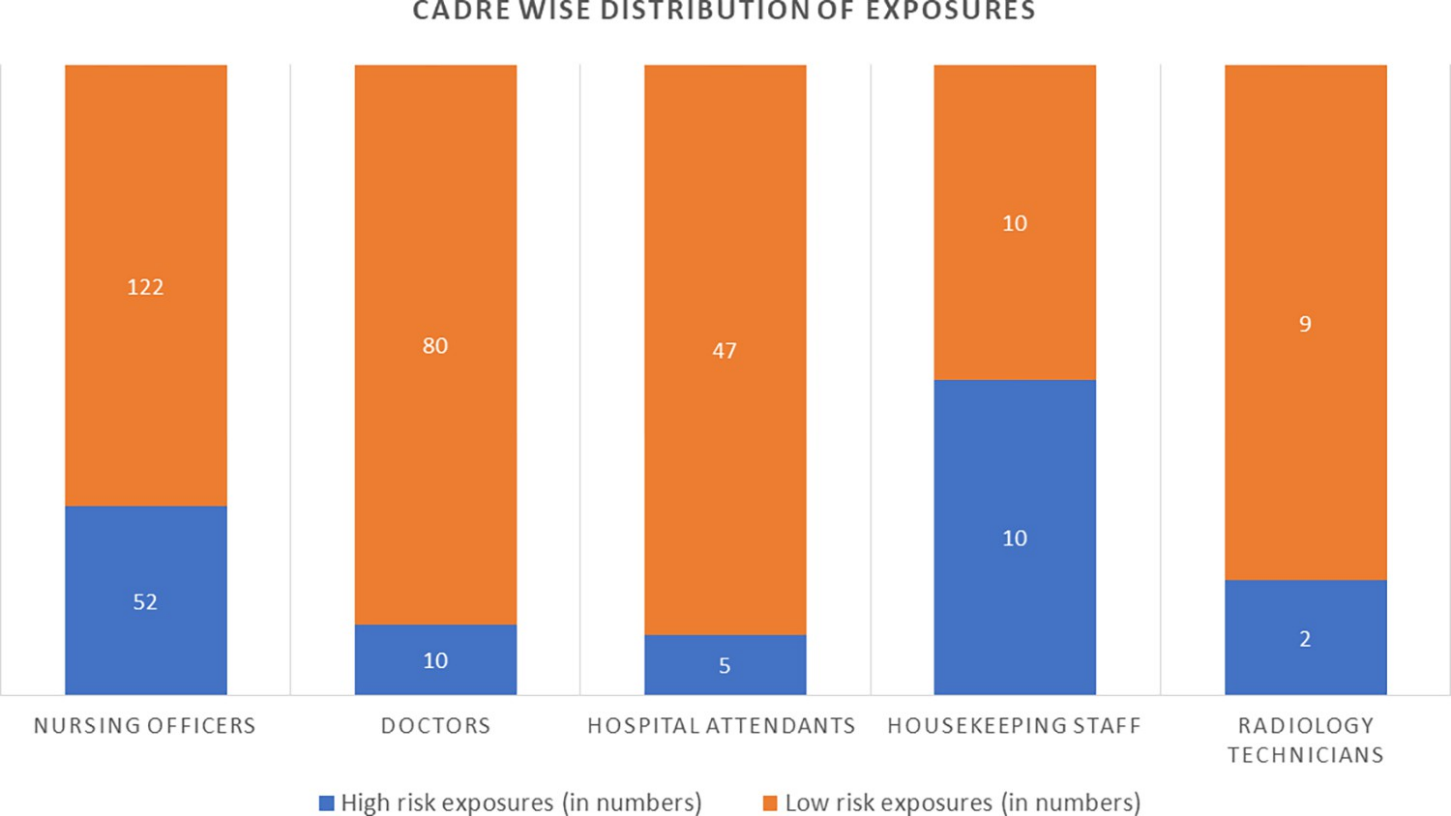
Cadre wise distribution of exposures. Number of low risk and high risk exposures occurred among various cadres of HCWs.

Cadre wise distribution of high and low-risk exposures revealed that maximum PPE breaches/exposures occurred in the category of nursing officers (n = 174, 50.1%) followed by doctors (n = 90, 26%), housekeeping staff (n = 52, 5.8%) and Radiodiagnosis technicians (n = 11, 3.1%) (Fig 2.) Out of the 268 low-risk exposures, maximum PPE breaches were observed in nursing officers (45.8%) followed by doctors (29.8%), housekeeping staff (21.2%), and technicians (3.5%). A similar trend was observed in high-risk exposures where out of the 79 exposures, 64.6% of the breaches in PPE were observed among nursing officers.

**Fig 3:**
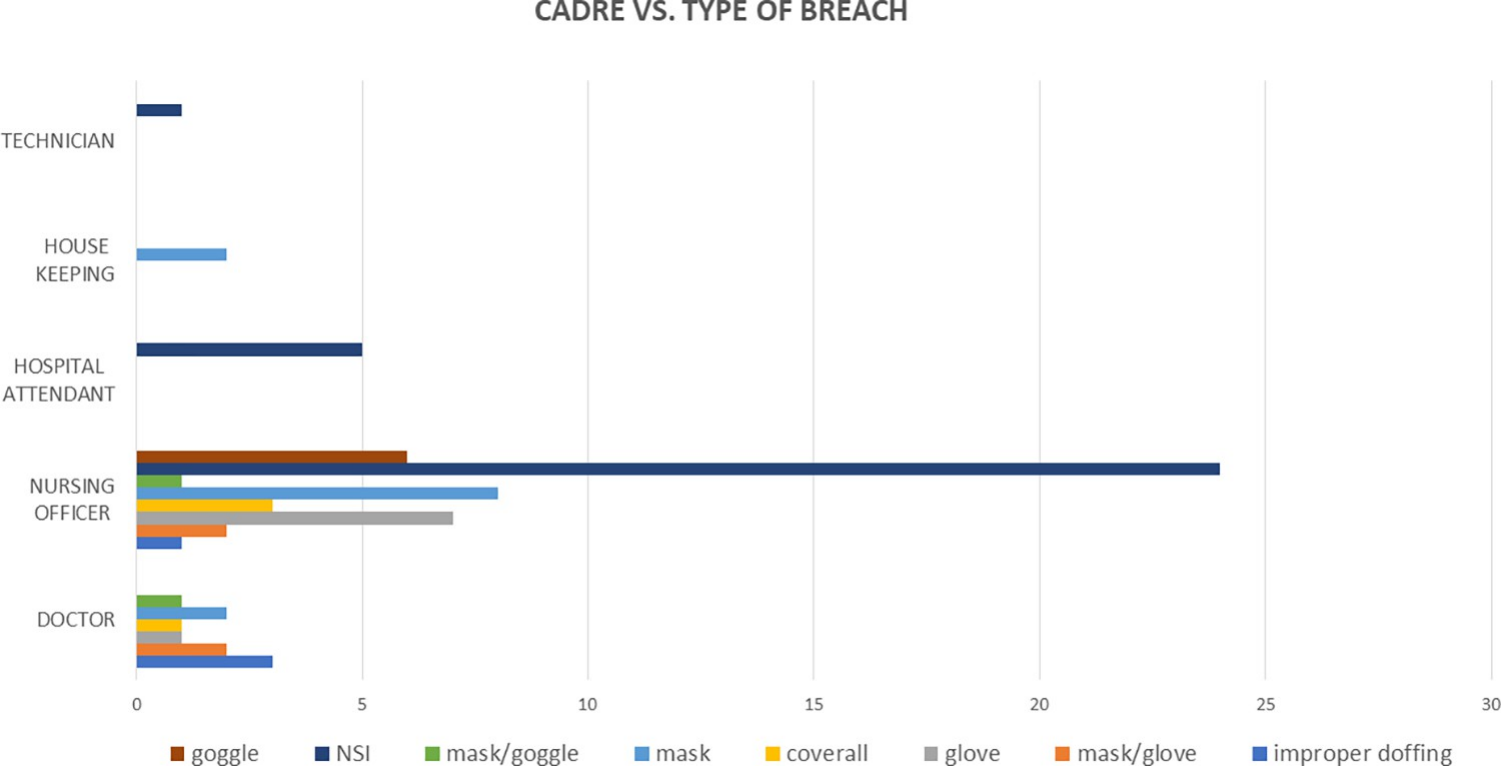
Breakdown of each cadre and the type of breach. (NSI: Needle stick injury).

Fig 3 shows the breakdown of cadres and the type of PPE breach. It was observed that among doctors, improper doffing of PPE was the most common. Amongst the nursing officers, hospital attendants and technicians, needle stick injury was the most common PPE breach whereas amongst the house keeping staff breach in the usage of masks was most common.

Exposures were also categorized according to the breaches observed with various components of PPE like gloves, masks, coverall gowns, goggles. These were classified into low or high-risk exposures as described in Table 1. In addition, improper doffing (steps missed/incorrectly done) of PPE was separately considered as a breach that can contribute to an exposure.

**Fig 4:**
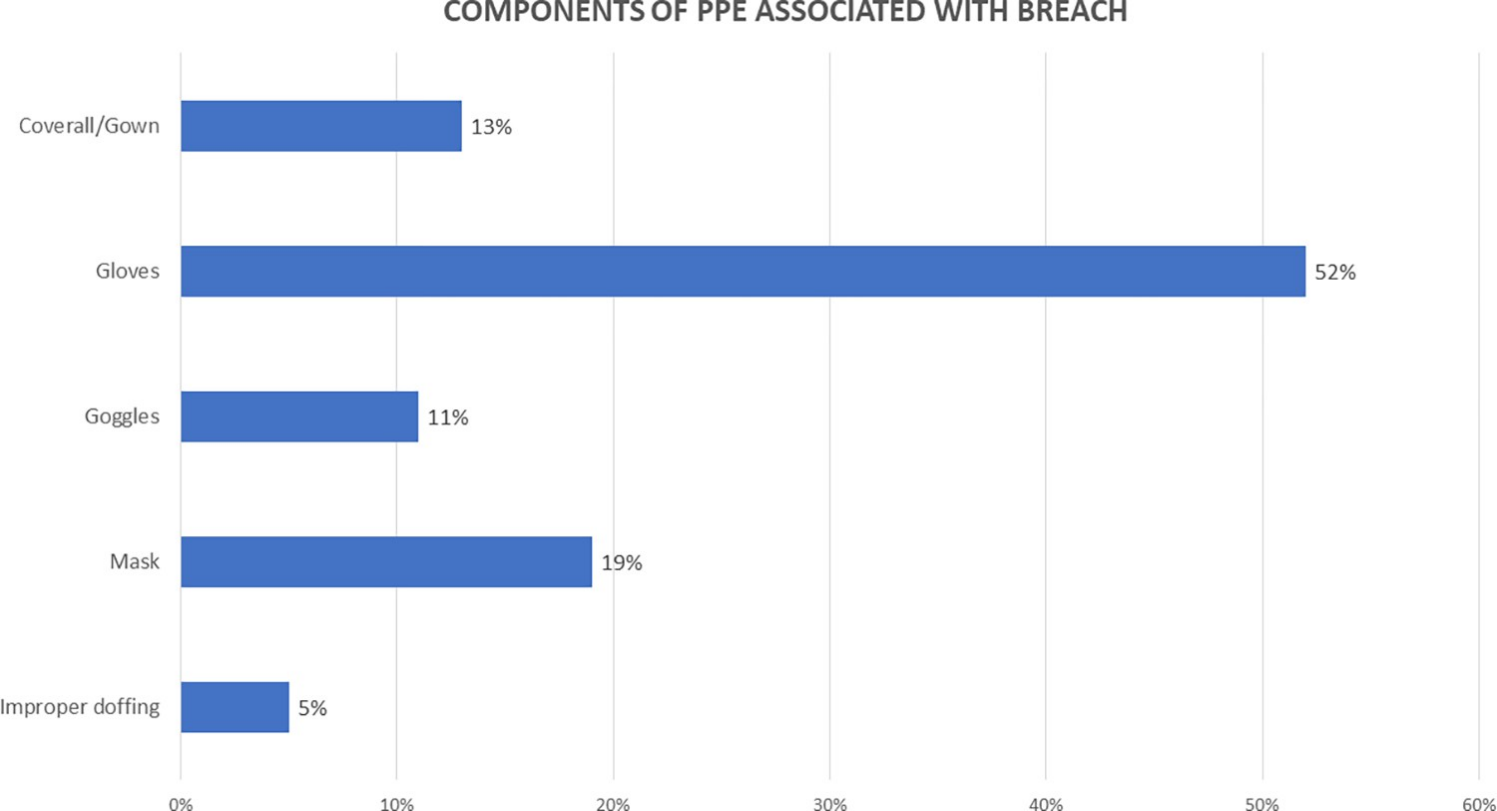
Components of PPE associated with breach.

As shown in Fig 4., the most common PPE breach reported was a tear in gloves (52%) coupled with non-adherence to hand hygiene following the tear/breach in gloves. This was followed by breaches in mask use (19%), coverall gowns (13%), goggles (11%), and improper doffing (5%).

### Correlation of PPE breach with subsequent COVID -19 positivity

Table 2 shows the distribution of PPE breaches associated with COVID-19 positivity. Among all, 12/79 (15.2%) high-risk exposures and 7/268 (2.6%) low-risk exposures were associated with COVID-19 positivity resulting in a cumulative positivity of 19/347 (5.4%). The most common single component of PPE breach associated with COVID-19 positivity was a mask related issue accounting for 7/19 (36.8%) of all the positive cases resulting from high and low-risk exposures followed by a breach in gloves and subsequent hand hygiene lapses 5/19 (26.4%), improper doffing 3/19 (15.8%), coverall gowns 2/19 (10.5%) and goggles 2/19 (10.5%). Collectively, non-mask related breaches accounted for the majority (63.2%) of the positive COVID-19 cases. Among the combined low- and high- risk exposures, improper doffing was associated with the highest COVID-19 positivity rate at 21.0%.

**Table 2:** Distribution of PPE breaches associated with COVID-19 positivity.

Type of PPE breach	Low-risk exposures (N = 268)	COVID-19 positivity (%) among low-risk exposures	High-risk exposures (N = 79)	COVID- 19 positivity (%) among high-risk exposures	Total exposures	Overall COVID-19 positivity (%) among all exposures
Gloves	124	3 (2.4%)	41	2 (4.8%)	165	5 (3.0%)
Mask	58	2 (3.4%)	15	5 (33.3%)	73	7 (9.6%)
Goggles	34	0 (0%)	9	2 (22.22%)	43	2 (4.6%)
Coverall	41	1 (2.4%)	10	1 (10.0%)	51	2 (3.9%)
Improper doffing	10	1(10.0%)	4	2 (50.0%)	14	3 (21.0%)
Total	268	7 (2.6%)	79	12 (15.2%)	347	19 (5.4%)

The odds ratio was found to be 2.07 (95% CI 0.79 to 5.46) which implies that getting COVID-19 positivity is almost two times higher amongst high-risk exposures as compared to the low-risk exposures.

## Discussion

Our findings suggest the best way for HCWs to prevent infection with SARS-CoV-2 is through training and demonstrated competency in putting on and removing PPE, also known as donning and doffing [11]. Our findings suggest strict adherence to the steps of donning and doffing will minimize the chances of exposure to infection. In our study, high-risk exposures contributed significantly fewer numbers among total exposures. This observation indicates good compliance by the HCWs to infection control practice (ICP) protocols as well as reflects stringent monitoring by the infection control team on-site to mitigate the risk.

Even after following all possible infection control guidelines, our findings illustrate that breaches may occur due to a number of reasons, resulting in variable levels of low- and high- risk exposures. Potential causes may include a combination of physical events and mental exhaustion resulting from work overload, improper self-care, or other situations outside the medical facilities [12].

It is worth noting that not all PPE breaches lead to high-risk exposures and not all high-risk exposures will lead to infection in the HCWs. The present study demonstrates a positivity rate of 15.2% and 2.8% for COVID-19 among the high-risk exposure and low-risk exposure groups respectively. The positivity among low-risk exposure groups may not be solely attributed to PPE violations, as there may be the possibility of unknown and unidentified exposures outside the work area. An analysis of COVID-19 transmission dynamics from a pediatric COVID- 19 care center demonstrated almost 70% (29 out of 42) of the positivity among HCWs without high-risk exposure in the hospital were classified as likely community-acquired [13]. A cross-sectional study of United States (US) HCW in 3 states analyzed higher odds (adjusted odds ratio [aOR], 3.5; 95% CI, 2.9–4.1;) of SARS-CoV-2 seropositivity among HCWs who had community exposures over those with presence of workplace factors, including workplace role (aOR, 1.1; 95% CI, 0.9–1.3), environment (aOR, 1.0; 95% CI, 0.8–1.3), or contact with patients with known COVID-19 (aOR, 1.1; 95% CI, 0.9–1.3) [14]. Infections occurring in low-risk groups can also be attributed to the risk perception by HCWs which may also play a role in exposures and transmission events. HCWs perceive coworkers or community exposures as less risky, which can increase transmission opportunities in non-clinical spaces since HCWs spend comparatively more time in confined and shared spaces such as nursing stations, physician workrooms, breakrooms, and conference rooms [15].

As described previously, positivity in the low-risk group was not totally associated with exposures at the workplace as the exposed individuals were not strictly quarantined and might had exposures in shared spaces or outside the workplace as there was no restriction for them to visit homes/community places during the off-duty hours as opposed to the high-risk exposure group. The positivity in the high-risk exposure group, on the other hand, can be linked to the workplace related exposures because these HCWs were strictly quarantined immediately after the exposure without further contact with unknown sources in the community or at shared workplaces. Even knowing this, the probability of contracting an infection through community exposures before coming into contact with a low or high-risk exposure at work cannot be completely eliminated. Hence, generating knowledge about the kinds of ICP breaches that are usually experienced by HCWs with its outcome is necessary and can guide upon future strategies to deal with the crisis.

The risk of transmission of SARS- CoV-2 may be related to the component of PPE used in the breach occurrence. In the present study, breaches in various components of PPE were observed. Amongst all, the most common breach observed was with the use of gloves, due to frequent tearing of gloves while doffing/working with them and due to needle stick injuries. However, it resulted in COVID- 19 positivity among 3.0% HCWs. A sizeable number of needle stick injury cases among the HCWs were observed, however, none of them resulted in COVID- 19 positivity. A breach in other components of PPE such as masking, coverall gown, goggles, and improper doffing was also observed. Chou et al [16] in their rapid living review on epidemiology and risk factors for COVID-19 in HCWs demonstrated the strongest evidence on risk factors was on PPE use and decreased infection risk. The association was most consistent for masks but was also observed for gloves, gowns, eye protection, and handwashing and emphasized the role of infection control training in mitigating the risk [16].

The transmission of SARS CoV-2 is principally through droplet infection and direct/indirect contact [6]. In our investigation, we observed that a breach in masking was associated with a positivity of 9.6% in all exposures and 33.3% in the high-risk exposures group among HCWs. A breach in goggles, was associated with a total and high-risk exposure COVID-19 positivity of 4.6% and 22.2%, respectively. This observation suggests that face protection is critical in preventing transmission. Another important observation is about the improper doffing of PPE that was associated with the highest COVID- 19 positivity rate at 21.0% among all exposures and 50% among high-risk exposures, however the numbers of observations were low. Thus, the need for adequate personal protective equipment, especially respiratory and eye protection is important during both high-risk procedures as well as routine patient care. At the same time, it is imperative that the HCWs follow the correct PPE doffing sequence and procedure to prevent getting significant exposures associated with COVID positivity.

Our study showed that breaches were scattered across staff groups, and it was reported that breaches were most common among the nursing officers probably because of their wider representation among all types of HCWs and continuous involvement in inpatient care.

Investigating common breaches identifies possible routes of infection, establishes recommendations for improving PPE design, and enables IPC teams to educate HCWs accordingly.

For a reduction in PPE breaches by the HCWs, further work needs to be done. ‘Eagle-eyed observer’ approach as suggested by Peng et al is an example that can detect and correct PPE breaches by health care workers [17]. Further attention can be given to the appropriate use of PPE during training besides the types needed. Increased compliance and reduced frequency of PPE breaches may be attained by improving PPE design with enhanced comfort [18].

Our study sheds light on the behavioral aspects/areas where HCWs can go wrong in their knowledge/perception and actual emphasis when it comes to adherence to PPE protocol and its proper use when working in a challenging environment. Many of the breaches happened because HCWs were in a rush to complete their duties within a certain amount of time while working in unfavorable settings, which is a unique issue in COVID- 19 wards. Continuous use of PPE has reported to significantly cause physical and neurological dysfunctions such as, headache/irritability, the urge to doff among the HCWs and causing nausea and difficulty in decision making [19].

Our findings will improve future HCW education and training to help them avoid typical errors such as poor wearing of masks and goggles, which can lead to easy removal/displacement, and inappropriate doffing of these and other PPE, such as gloves, which can lead to contamination of hands. Hand hygiene’s importance can be emphasized even further by providing hands-on training in this crucial area. It also prompts a thorough examination of concerns relating to the quality of PPE used in the wards (such as a loose head harness or gloves made of inferior material that rip frequently) and helping the hospital administration in selecting the most appropriate products for safe use.

### Limitations

The passive nature of collection of information is one of the major limitations of the study. However, data on PPE breaches in subsequent waves will add to our knowledge in this area. To our knowledge this is the first study of its kind in India which assesses the type of PPE breaches among HCWs working in COVID-19 areas and subsequent positivity associated with the breach and our detailed analysis adds unique insights. Furthermore, to minimize the reporting bias, information collection and verification was done at multiple levels including immediately informing the infection control nurse (ICN) posted in the COVID-19 area and categorizing the breach as high- risk or low-risk followed by further verification by the Hospital infection control officer.

## Conclusion

HCWs being the frontline staff in handling the COVID-19 crisis are at a higher risk of acquiring infection. Appropriate use of PPE by HCWs is vital for their protection. Additionally, training on the appropriate use of PPE and other ICP measures, supervision and monitoring of ICPs can enhance the effectiveness of these interventions. However, despite all mechanisms in place, a breach in the PPE may occur while managing COVID-19 patients due to physical and mental exhaustion among HCWs resulting from work overload. Early identification and appropriate management of HCWs with both low- and high-risk exposures can prevent transmission to other hospital staff, saving resources and workforce.

## Supporting information

S1 File (PDF)
