# Supplementary material for: A retrospective observational insight into COVID-19 exposures resulting from personal protective equipment (PPE) breaches
Source: PLoS One. 2022 May 17;17(5):e0268582. doi: 10.1371/journal.pone.0268582 (PMC9113574; doi:10.1371/journal.pone.0268582)
Supplement: S1 File — (PDF) [file pone.0268582.s001.pdf]

Hospital Infection Control Committee, AIIMS Raipur

Recommendations for use of Masks and other PPE at AIIMS Raipur in relation to COVID-19

Version 1.0

Dated – 11th April 2020

In the context of current situation of COVID – 19 pandemic and preparing ourselves to deal with it, it is imperative to use Personal Protective Equipment (PPE) appropriately as well as judiciously in health care settings. It also must be noted that, overall, there is a shortage in supply of these PPE in our country.

In view of this, guidelines have been issued herewith for rational use of masks and other PPE by the health care workers of AIIMS Raipur. The guidelines are based upon the WHO and Ministry of Health and Family Welfare, Government of India's recommendations as available till date.

The inputs from COVID 19 Preparedness Committee, AIIMS-Raipur are also incorporated in the document.

All heath care workers are requested to adhere to the guidelines and use only the recommended PPE to protect themselves, others and the hospital environment.

A. Recommendations for use of PPE in COVID-19 Designated areas of the hospital

Note: Health care staff posted in this area should wear dedicated cloths provided by the hospital

Area	Target HCW/ personnel	Activity	Surgical mask	N 95 Mask	Gloves	Gown/ Coverall	Head cap	Goggles	Shoe covers	Hood
Help desk	Any HCW	Patient enquiry/ Interview and documentation		√*	√					
	Patient	Suspected of COVID	√							
Registration counter	Any HCW	Computer entry/ Preparing OPD/IPD registration slip		√*	√					
Triage or screening zone	Doctors/ Nursing officers	History taking, recording of vitals, data entry		√*	√					
	Doctors/ Nursing officers	Clinical examination of suspected patient (No aerosol generating procedures are done)		√*	√	Reusable gown	√			
	Hospital attendants/ HCW	Patient shifting		√*	√	Reusable gown	√			
Sample collection area	Doctors/ Nursing officers/ Technicians	Respiratory/ other collection of COVID suspected/confirmed cases		√	√ (Double)	Coverall OR (Gown with plastic apron)	√	√	√	√
	Attendants/ HCW	Sample transport to the laboratory	√		√					

*N95 masks used in these places should be re-used as per the Re-use policy of the Institute.

<table>
<tr><th>Area</th><th>Target HCW/ personnel</th><th>Activity</th><th>Surgical mask</th><th>N 95 Mask</th><th>Gloves</th><th>Gown/ coverall</th><th>Head cap</th><th>Goggles</th><th>Shoe covers</th><th>Hood</th></tr>
<tr><td rowspan="4">Isolation ward/room/ ICU/ HDU holding suspected or confirmed COVID – 19 patients</td><td>Any HCW</td><td>• Clinical management of suspected/ confirmed cases irrespective of involvement in aerosol generating procedures
• Dead body packaging</td><td></td><td>√</td><td>√ (double)</td><td>Coverall</td><td>√</td><td>√ (with face shield preferably)</td><td>√</td><td>√</td></tr>
<tr><td>Visitor / patient relative</td><td>Visiting the ward/ taking care of admitted patient (restricted entry)</td><td></td><td>√</td><td>√</td><td>Reusable gown</td><td></td><td></td><td></td><td></td></tr>
<tr><td rowspan="2">HCW involved in patient shifting/ transport</td><td>Transport of sick patients</td><td></td><td>√</td><td>√</td><td>Reusable gown</td><td>√</td><td>√</td><td>√</td><td>√</td></tr>
<tr><td>Transport of dead bodies (packed and disinfected)</td><td>√</td><td></td><td>√</td><td></td><td></td><td></td><td></td><td></td></tr>
<tr><td>Ancillary areas to isolation room/ward</td><td>Any HCW</td><td>Supporting staff not involved in direct patient interaction/ patient care (e.g managing the data/ stores/ inventory etc)</td><td>√</td><td></td><td></td><td></td><td>√</td><td></td><td></td><td></td></tr>
<tr><td>Corridors/ other areas of transit</td><td>Any HCW</td><td>Any activity that does not involve contact with patient</td><td>√</td><td></td><td colspan="7" rowspan="2">• Spatial distancing of one meter to be ensured
• Hand hygiene should be followed in case of contact with fomites and at the time of exit.</td></tr>
<tr><td>Any area</td><td>Patients</td><td>With respiratory symptoms/ suspects/ confirmed</td><td>√</td><td></td></tr>
</table>

B. Recommendations for Non COVID areas of the hospital

Area	Target HCW/ personnel	Activity	Surgical mask	N 95 Mask	Gloves	Gown/ Coverall	Head cap	Goggles	Shoe covers	Hood
ICUs/ HDUs/ Red Zones/ Emergency OTs/ Procedures (on non COVID patients)	Health care workers involved in direct patient care	Attending to seriously ill patients or operating/assisting surgeries	√	√ (If AGP / SARI patients)	√ (sterile)	Sterile Surgical gown + plastic apron (optional)	√	√ (If AGP/ SARI patients)	√	
Emergency OTs/ Procedures on COVID confirmed cases	Health care workers directly involved in the procedures	Performing/ assisting the surgeries including anaesthetist and scrub nurses		√	√ (sterile double pair)	**Sterile surgical gown to be worn over the coverall**	√	√ (face shield if available)	√	√
Trauma and Emergency areas	Health care workers	Attending any emergency case		√	√	Surgical Gown	√	√ (If AGP/ SARI patients)		
Dental procedure rooms	Health care workers	Dental procedures on emergency patients	√	√ (If AGP)	√ (sterile)	Surgical gown + plastic apron (optional)	√	√ (If AGP)		
Other Wards/ Indoor settings	Health care workers	Attending routine patients	√	√ (if AGP)	Other PPE should be worn according to standard precautions and individual risk assessment.					
	Patients/ HCWs	With respiratory symptoms	√	• Distancing of one meter to be ensured<br>• Hand hygiene should be followed in case of contact with fomites and at the time of exit.						
OPDs	Health care workers	Attending routine patients	√	√ (If AGP)	Other PPE should be worn according to standard precautions and individual risk assessment					

	Patients/HCWs	With respiratory symptoms	√	• Distancing of one meter to be ensured<br>• Hand hygiene should be followed in case of contact with fomites and at the time of exit

AGP – Aerosol generating procedures; SARI - Severe acute respiratory illness

C. Recommendations for other supportive/ ancillary services

**Area**	**Target HCW/ personnel**	**Activity**	**Surgical mask**	**N 95 Mask**	**Gloves**	**Gown/ coverall**	**Head cap**	**Goggles**	**Shoe covers**	**Hood**
**Laboratory services**										
Sample collection area (in screening zone or isolation ward)	Doctor/ Nursing officer/ Technician	Respiratory and other sample collection from COIVID suspected/ confirmed patient		√	√	Coverall OR Gown with plastic apron	√	√	√	√
Sample transport	Laboratory/ hospital attendant	Sample Transportation from COVID as well as Non COVID area	√		√					
All laboratories	Doctor/ Research fellow/ Technician/ Nursing officer/ attendant	Processing of respiratory samples from COVID patients		√	√	Coverall OR Gown with plastic apron	√	√	√	√
		Processing on any other sample from COVID patients that involve aerosol generation		√	√	Plastic apron				
		Collection, transport and processing of any sample from Non COVID patients	√		√					

**Housekeeping/ Sanitation/ Laundry/ BMW handlers**										
**Area**	**Target HCW/ personnel**	**Activity**	**Surgical mask**	**N 95 Mask**	**Gloves (Heavy duty)**	**Gown* / Coverall**	**Head cap**	**Goggles**	**Shoe covers**	**Hood**
Help desk/ Screening zone/ waiting area/ registration counter at COVID- 19 area	Housekeeping staff	Cleaning of high touch surfaces/ floors/ Washrooms etc		√	√	Gown*		Only if splashes expected	Gum boots	
Isolation ward/ room/ICU/HDU at COVID-19 area	Housekeeping staff	Cleaning of high touch surfaces/ floor / washrooms etc		√	√ (Discard after use)	Coverall	√	√	√	√
Non COVID – 19 areas	Housekeeping staff	Cleaning of high touch surfaces/ floors etc	√		√	Plastic Apron only			Gum boots	
Any area	Housekeeping staff	Spraying of disinfectant in premises	√		√	Gown*	√	√	Gum Boots	
COVID – 19 areas	BMW handlers	Collection and transport of BMW		√	√ (Discard after use)	Coverall	√	√	Gum Boots	
COVID – 19 areas	Laundry persons	Cleaning and disinfection of soiled linen		√	√ (Discard after use)	Coverall	√	√	Gum Boots	

***Gown should be full sleeved and water resistant gown. If not water resistant, use plastic apron along with gown.**

Area	Target HCW/ personnel	Activity	Surgical mask	N 95 Mask	Gloves	Gown / Coverall	Head cap	Goggles	Shoe covers	Hood
Ambulance	HCWs/attendants	Transporting COVID patients not on assisted ventilation		√	√	Reusable gown				
	HCWs/ Attendants	Management of SARI patients while transporting		√	√	Coverall	√	√	√	√
	Drivers	Driving the ambulance and no contact with patient	√							
	Driver	Driving the ambulance and assisting with loading or unloading patient	√		√	Reusable gown				
	Cleaners/ Housekeeping staff	Cleaning and disinfection of Ambulance	√		√ (Heavy duty gloves – discard after use)	Water resistant gown or with plastic apron	√	√	Gum boots	
	Patients	While being transported to hospital	√							

<table>
<tr><th>Area</th><th>Target HCW/ personnel</th><th>Activity</th><th>Surgical mask</th><th>N 95 Mask</th><th>Gloves</th><th>Gown/ Coverall</th><th>Head cap</th><th>Goggles</th><th>Shoe covers</th><th>Hood</th></tr>
<tr><td rowspan="2">Mortuary</td><td rowspan="2">Any Health care worker</td><td>Dead body handling of COVID positive patient</td><td></td><td>√</td><td>√</td><td>Coverall</td><td colspan="4">• No aerosol generating procedures should be allowed
• No embalming</td></tr>
<tr><td>While performing autopsy (No Post-mortem unless until specified)</td><td></td><td>√</td><td>√</td><td>Coverall</td><td>√</td><td>√</td><td>√</td><td>√</td></tr>
<tr><td rowspan="3">Radiodiagnosis</td><td rowspan="3">Doctors/ Technicians</td><td>Routine work</td><td>√</td><td></td><td></td><td></td><td></td><td></td><td></td><td></td></tr>
<tr><td>Any procedure involving close contact with patients having respiratory symptoms</td><td>√</td><td>√ (if AGP)</td><td>√</td><td>Reusable gown</td><td></td><td></td><td></td><td></td></tr>
<tr><td>Any procedure involving close contact with the patient in COVID ward/ICU/HDU</td><td colspan="8">Follow full PPE protocol of COVID – 19 area if involved in radiological examinations in such areas</td></tr>
<tr><td>Amrit Pharmacy</td><td>Pharmacist</td><td>Drug dispensing</td><td>√</td><td></td><td></td><td></td><td></td><td></td><td></td><td></td></tr>
<tr><td>CSSD</td><td>Technicians</td><td>Handling linen or instruments (cleaned and pre-treated) from COVID-19 patients</td><td>√</td><td></td><td>√</td><td></td><td></td><td></td><td></td><td></td></tr>
<tr><td>Security at COVID -19 areas</td><td>Security personnel</td><td>No direct contact with patient</td><td>√</td><td colspan="7">• Maintain distance of 1 meter from suspected patient
• Perform Frequent hand hygiene
• Follow PPE protocol of COVID – 19 area if entering into/ assisting/ transporting patients in isolation or screening areas</td></tr>
</table>

Security at Non COVID-19 areas	Security personnel	No direct contact with patient	• NO PPE required<br>• Maintain distance of 1 meter from suspected patient<br>• Only personnel having respiratory symptoms should wear triple layered mask or cloth mask
Academic section, Administrative, Finance offices/ Engineering	All staff	No direct/ indirect patient contact	• NO PPE required.<br>• Only personnel having respiratory symptoms should wear triple layered / cloth mask.<br>• The staff should not venture into COVID-19 treatment area. If required to enter, follow the PPE protocol of that area.<br>• HCW visiting these places after hospital visit should remove their soiled PPE and perform Hand hygiene at the entrance.

Precautions that must be followed while using masks

- PPEs are not alternative to basic preventive public health measures such as hand hygiene, respiratory etiquettes which must be followed at all times.
- Masks should be worn securely with proper fit on the face.
- N95 masks should be seal checked after wearing.
- Avoid touching outer surface of the mask while in use.
- If accidentally touched – perform hand hygiene.
- Do not re- use the single- use mask.
- Replace the masks if become damp/wet or if used for >6 hours
- While removing open the lower string first.
- Remove the mask holding only the lace/string and not the outer surface.
- After removal discard the mask in yellow waste bin.
- Perform hand hygiene after mask removal.

Note - Wearing masks when they are not indicated may cause unnecessary cost and procurement burden and create false sense of security that can lead to neglecting other essential preventive measures.
